# Design and evaluation of electron beam energy degraders for breast boost irradiation

**DOI:** 10.1186/s13014-016-0686-7

**Published:** 2016-08-31

**Authors:** Jong In Park, Sung Whan Ha, Jung-in Kim, Hyunseok Lee, Jaegi Lee, Il Han Kim, Sung-Joon Ye

**Affiliations:** 1 Department of Transdisciplinary Studies, Program in Biomedical Radiation Sciences, Seoul National University Graduate School of Convergence Science and Technology, Seoul, 151-742 Korea; 2 Institute of Radiation Medicine, Seoul National University Medical Research Center, Seoul, Korea; 3 Department of Radiation Oncology, Seoul National University Hospital, Seoul, Korea; 4 Interdisciplinary Program in Radiation Applied Life Science, Seoul National University College of Medicine, Seoul, Korea; 5 Advanced Institutes of Convergence Technology, Seoul National University, Suwon, Korea

**Keywords:** Electron energy degrader, Breast boost irradiation, Monte Carlo simulation, Linear accelerator

## Abstract

**Background:**

For breast cancer patients who require electron boost energies between 6 and 9 MeV, an energy degraders (ED) in the 9 MeV beamline was specially designed and manufactured to increase the skin dose of 6 MeV and to reduce the penetration depth of 9 MeV beams.

**Methods:**

We used Monte Carlo (MC) techniques as a guide in the design of ED for use with linear accelerators. In order to satisfy percent depth dose (PDD) characteristics and dose profile uniformity in water, the shape and thickness of Lucite® ED in the 9 MeV beamline was iteratively optimized and then manufactured. The ED geometry consists of a truncated cone attached on top of a plane plate, with total central thickness of 1.0 cm. The ED was placed on the lower most scraper of the electron applicator. The PDDs, profiles, and output factors were measured in water to validate the MC-based design.

**Results:**

Skin doses with the EDs increased by 8–9 %, compared to those of the 9 MeV beam. The outputs with the EDs were 0.882 and 0.972 for 10 × 10 and 15 × 15 cm^2^ cones, respectively, as compared to that of a conventional 9 MeV beam for a 10 × 10 cm^2^ cone. The X-ray contamination remained less than 1.5 %. In-vivo measurements were also performed for three breast boost patients and showed close agreement with expected values.

**Conclusions:**

The optimally designed ED in the 9 MeV beamline provides breast conserving patients with a new energy option of 7 MeV for boost of the shallow tumor bed. It would be an alternative to bolus and thus eliminate inconvenience and concern about the daily variation of bolus setup.

## Background

Electron boost irradiation for breast cancer patients is routinely practiced in modern radiotherapy clinics. Electron beams are capable of covering planning target volumes (PTV) with an appropriate prescription dose while sparing the underlying critical structures [1]. Electron beams are advantageous over photon beams for breast boost irradiation due to more effective sparing of distally located organs at risk (OAR), and the ability to deliver a fairly uniform dose to the target volume [2]. In general a depth of 90 % or 80 % of dose maximum is the therapeutic range of electron beam [1]. Thus, in order to allow personalized treatments with an optimal energy, fine energy spacing of electron beams of commercial linear accelerators (LINAC) is necessary. However, with an electron beam of 6 or 9 MeV, it is difficult for the skin dose to reach 90 % of the dose maximum while at the same time having the therapeutic range located between the distal end of the target volume and the proximal part of the OAR.

In order to increase the skin dose while retaining high dose fall-off beyond the depth of dose maximum (*d*_max_), a thin tantalum wire mesh placed on the patient’s skin has been proposed [3, 4]. A similar energy control has also been achieved through the use of a high-density metal foil bolus [5]. A water equivalent bolus has been widely used during part of the treatment to raise the skin dose and to reduce the energy or therapeutic range [6, 7]. However, daily setup variation with the bolus may cause uncertainties in the dose delivered to the target volume.

An advanced technique using a prototype electron multi-leaf collimator (eMLC) to create narrow and segmented beams has been used to modulate electron energy without the setup variation [8–15]. Modulation of adjacent narrow segments with the eMLC enhances the skin dose while sparing surrounding normal tissues [16]. In addition to the eMLC, the few leaf electron collimator (FLEC) has also been used for an alternative to bolus for boost treatment of tumor bed in breast cancer [17, 18]. Although this technique is promising, it requires an add-on to the LINAC (i.e., the eMLC) and is still a kind of prototype. Options for energy spacing remain restricted in clinical situations.

It was well known that a spoiler made of low-atomic number (Z) material could enhance the skin dose for electron treatments with beam energies between 6 and 12 MeV while limiting the penetration depths above OARs [19]. In this study we produced an electron beam spoiler without modifying components of the LINAC. The spoiler can provide a therapeutic range between 6 and 9 MeV, and is thus termed an energy degrader (ED). In addition, it could eliminate concern about the daily variation of bolus setup. We performed Monte Carlo (MC) simulations to optimize the design of the ED system by calculating beam characteristics such as depth doses, uniformity, dose rates, and bremsstrahlung contamination. Prior to clinical use, rigorous measurements were taken to validate the MC-based optimization.

## Methods

### Clinical linear accelerator

The medical LINAC used in this study was Varian Trilogy (Varian Oncology Systems, Palo Alto, CA). Six electron energies (4, 6, 9, 12, 16 and 20 MeV) were available, and fields were shaped with open walled applicators consisting of three scrapers and matched scattering foils. A final field-defining Cerrobend cutout was placed on the lowermost scraper. The electron applicators have a nominal source-to-end of applicator distance of 95 cm. This indicates a 5 cm air-gap between the applicator end and the standard source-to-surface distance (SSD) = 100 cm plane. The beams investigated in this study were the standard 9 MeV beam and an ED-moderated beam in the standard 9 MeV beamline, hereafter often denoted 7 MeV beam since an ED of 1 cm water-equivalent thickness moderates electron energy by 2 MeV approximately. They were collimated by 10 × 10 and 15 × 15 cm^2^ applicators.

### Monte Carlo simulation

The geometry and compositions of the primary collimator, vacuum window, scattering foil, monitoring ion-chamber, mirror, movable jaws, etc. for the 9 MeV electron mode, and the 10 × 10 and 15 × 15 cm^2^ applicators and their scrapers were obtained from information supplied by the manufacturer. The construction details of the LINAC treatment head were also provided by the manufacturer, whereas the energy degraders were built in our laboratory. The uppermost and middle scrapers of the electron applicator were modeled using the EGSnrc/BEAMnrc component module (CM) APPLICAT [20]. The lowermost scraper was modeled by PYRAMIDS to insert the ED into the cutout insert, and the ED was modeled by CONESTAK. Our EGSnrc/BEAMnrc simulations consisted of two major steps. The first step involved adjusting the primary electron beam parameters of our LINAC to match the 9 MeV beam data measured. In the second step, these beam parameters were then used to compute dose distributions with various different designs of energy degraders, and thereby provided guidance for the manufacturing of optimized EDs. The EGSnrc/DOSXYZnrc code was used to calculate dose distributions in a 30 × 30 × 30 cm^3^ water phantom at 100 cm SSD, irradiated by the 99.9 cm SSD phase space determined in the previous simulations [21]. The doses in the water phantom were scored in voxels of 0.5 cm (width) × 0.5 cm (length) × 0.2 cm (depth). In the EGSnrc/BEAMnrc simulations, the initial number of histories was 3.0 × 10^8^ particles emitting from the vacuum window. Approximately 1.0 × 10^8^ particles were scored in the phase space at 99.9 cm SSD with an air slab of 0.1 cm thickness. In the EGSnrc/DOSXYZnrc simulations, 3.0 × 10^8^ particles were sampled from the 99.9 cm SSD phase space file as a source, yielding statistical uncertainties of < ±2 % along the cross-beam profiles of −8 to +8 cm for a 10 × 10 cm^2^ field, and along the cross-beam profiles of −9 cm to +9 cm for 15 × 15 cm^2^. Such a number of particles required the phase space file to be recycled 1 up to three times. Statistical uncertainties in depth doses along the central axis were less than ±1 % except in the bremsstrahlung tails (< ±2.5 %). In accordance with previously published papers [20, 22–28], ECUT (the energy cut-off for electron transport) was set to 700 keV for the EGSnrc/BEAMnrc and EGSnrc/DOSXYZnrc simulations. PCUT (the energy cut-off photon transport) was set to 10 keV for both simulations. Below these cut-off energies the kinetic energy of the particle was considered to be absorbed locally.

To find the parameters of the electron beam incident on the vacuum window, we followed published procedures by matching our calculated depth doses and cross-beam profiles to our measurements in a water phantom [22, 29, 30]. We started with published beam parameters from a model of a linear accelerator identical to ours, and made small fine-tuning adjustments until the best match was found [22, 23, 28, 31]. Measured cross-beam dose profiles were found to be symmetric and we thus used a normally incident beam with no lateral shift. Varied parameters of the parallel circular beam included the full width at half maximum (FWHM) of the Gaussian radial distribution and the energy of the incident electron source. Tuning of incident electron parameters was performed by comparing calculated and measured relative central axis depth doses and cross-beam profiles at 100 cm SSD for 10 × 10 and 15 × 15 cm^2^ ones.

We investigated beam parameters with incident energies of 9.75, 9.85, 9.95 and 10.05 MeV having Gaussian radial distributions with FWHM of 0.12, 0.13, 0.15 cm. The beam parameters that yielded the closest agreement between simulations and measurements were considered as the best estimate of the actual beam parameters and used for all subsequent calculations.

### Design of the energy degrader

**Fig. 1:**
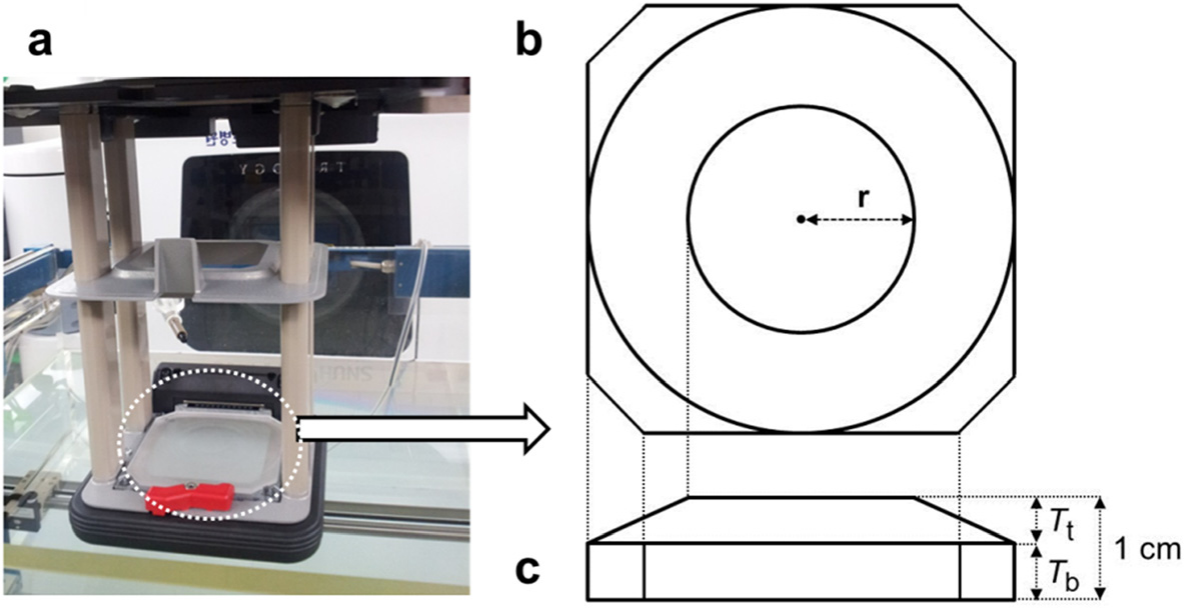
Construction of energy degrader. Construction of the energy degrader (ED) for 7 MeV beam investigated using the Monte Carlo simulations. The shape of ED was a truncated cone attached on the plane plate and the central thickness of ED was 1.0 cm. **a** A picture of the optimized energy degrader placed on the plane plate of the lowermost scraper for 10 × 10 cm^2^ cone. It was secured in the cutout insert. **b** The top view of ED; the parameter *r* is the radius of circle on the upper layer. **c** The side view of ED; *T*_*t*_ is the thickness for upper layer and *T*_*b*_ is the thickness of bottom layer. Ratio of two layer’s thickness varied with design-to-design

It is possible to change the energy of the electron beam by tuning the current of the bending magnet from the standard 6 or 9 MeV [32]. However, this procedure requires the standard energy to be replaced with a different energy. In contrast, we chose to insert an ED into an electron applicator. Once the source parameters of the 9 MeV beam were determined, various EDs inserted into an applicator of either 10 × 10 or 15 × 15 cm^2^ cone were tested using MC simulations. We limited our investigation to the EDs placed on the lowermost scraper, which was the case to minimize scattered doses outside of treatment field (Fig. 1a). With an ED in place, dose uniformity at depths could be worse than that of electron beams with no ED (ED-P). Thus one of our design goals was to achieve a uniform dose region at treatment depths. A simple double-layer approach was chosen for the ED design as shown in Fig. 1b-c. Our investigations included six different ED designs for both 10 × 10 and 15 × 15 cm^2^ cones, as summarized in Table 1. Five of the ED designs incorporated a truncated cone attached on top of the plane plate, where the bottom layer was a plate shape and the edge of the upper layer was carved to improve uniformity as shown in Fig. 1c. As shown in Table 1, the radius of the top layer *r*, top layer thickness *T*_t_, and bottom layer thickness *T*_b_ were chosen to achieve the best possible uniformity at *d*_max_ of the cross-beam dose profile. The total thickness of the Lucite® (*T*_t_ + *T*_b_) was 1 cm to reduce electron energy by approximately by 2 MeV. The radius of the top plate was fixed as an empirically chosen value (2 cm) when simulating the 10 × 10 cm^2^ field because varying only *T*_*t*_ or *T*_*b*_ was sufficient for improving the profile uniformity. In addition, a simple plate (ED-P) was simulated for comparison. Output factors were calculated to quantify the output loss by scattering with the EDs and compared with measured values. The reference configuration of the output factor was a 10 × 10 cm^2^ open applicator field with the water phantom at a nominal SSD of 100 cm. The output factor for a given field with the ED was calculated by taking the ratio of the maximum calculated dose in that configuration to the maximum calculated dose in the reference configuration. Output factors with optimized EDs were calculated for 10 × 10 cm^2^ and 15 × 15 cm^2^ applicators.

**Table 1:** Parameterized values to optimize the energy degrader. Parameterized values to optimize the energy degrader for 10 × 10 cm^2^ and 15 × 15 cm^2^ cone size. *r* is the radius of top layer. *T*_t_ is the top layer thickness. *T*_b_ is the bottom layer thickness. *T*_t_ + *T*_b_ is equal to 1.0 cm

10 × 10 cm^2^ cone				15 × 15 cm^2^ cone			
	*r*	*T*_t_	*T*_b_		*r*	*T*_t_	*T*_b_
ED-P^a^	-	-	1.0	ED-P^a^	-	-	1.0
ED-1	2	0.7	0.3	ED-1	2	0.6	0.4
ED-2	2	0.6	0.4	ED-2	3	0.6	0.4
ED-3	2	0.5	0.5	ED-3	6	`	0.4
**ED**-**4**	2	0.4	0.6	**ED**-**4**	4	0.3	0.7
ED-5	2	0.3	0.7	ED-5	6	0.3	0.7

^a^ED-P is a Lucite® slab plate, thickness of which is1.0 cm

Highlighted bold ED-4 was selected as an optimal design

Lucite®, also known as polymethly methacrylate (PMMA), was chosen as the selected material for the ED. Lucite® is a water-equivalent material with a relatively low Z, which is expected to decrease electron energy by approximately 2 MeV per cm and to cause relatively low X-ray contamination [33].

### Measurement with ion-chamber

Relative central-axis depth dose and cross-beam dose profiles for the standard 9 MeV electron beam were measured in water using a CC13 thimble ion-chamber (PTW, Freiburg, Germany) in a Blue phantom (IBA dosimetry, Louvain-la-Neuve, Belgium) at 100 cm SSD. These measured data were acquired and analysed with data acquisition software (OmniPro Accept version 7.1A). The electron beams were perpendicular to the phantom within the accuracy of machine setup. Steps of 0.5 mm with a 1 s dwell time at each measurement position were used. The depth of dose maximum, along with 90, 80 and 50 % of dose maximum are represented by *d*_max_, *R*_90_, *R*_80_, and *R*_50_. The practical range, which is the depth where the tangent to the linear portion of the central-axis depth-dose curve intersects the extrapolated X-ray contamination, is represented by *R*_p._ The most probable energy (*E*_0_) of the incident electron beam was calculated. All measurements and corrections in this study were done in accordance with the recommendations of the AAPM TG-51 protocol and TG-25 report [34, 35].

Output factors were measured with a CC13 ion-chamber by placing the effective point of measurement at the predetermined measured position of *d*_max_ for the given and reference fields. The TG-51 protocol recommends the use of a parallel plate chamber for electron beams with *R*_50_ less than or equal to 4.3 cm [34]. The thimble ionization chamber (Scanditronix Medical AB, Uppsala, Sweden) was used to measure the output factors for electron beams of energy less than 10 MeV [36–38]. The thimble ionization chamber has the geometry almost identical to a CC13 ion-chamber. Several other studies also used the cylindrical chambers to measure dosimetry data for low energy electron beams [32, 39]. The reference field for output factor measurements was an open field with the 10 × 10 cm^2^ applicator. In this study, a 10 × 10 cm^2^ applicator defines a field of 10 × 10 cm^2^ at SSD = 100 cm. The diameters of the Cerrobend cutouts were 3, 4, 5, 6, 7, 8 and 9 cm for the 10 × 10 cm^2^ cone, and 8, 9, 10 and 12 cm for 15 × 15 cm^2^. Values of cutout factor were listed along the ratio of area to perimeter (A/P) of the equivalent square field for a circular cutout (Table 5).

### In-vivo dosimetry

The new 7 MeV beam was implemented to our in-house MU calculation software. Dose characteristics were investigated from SSD = 90 cm to 110 cm. This software was based on AAPM TG-71 protocol [40]. The reference dosimetry following the AAPM TG-51 protocol was performed to calibrate the OSLDs [34, 41]. The PTW30013 Farmer type chamber (PTW, Freiburg, Germany) was used for this purpose. It was reported that the cylindrical PTW Farmer chamber and the parallel plate PTW Roos chamber agreed within 1 % for the 4 and 6 MeV energies, and within 0.5 % for the 9 and 12 MeV energies [42].

**Fig. 2:**
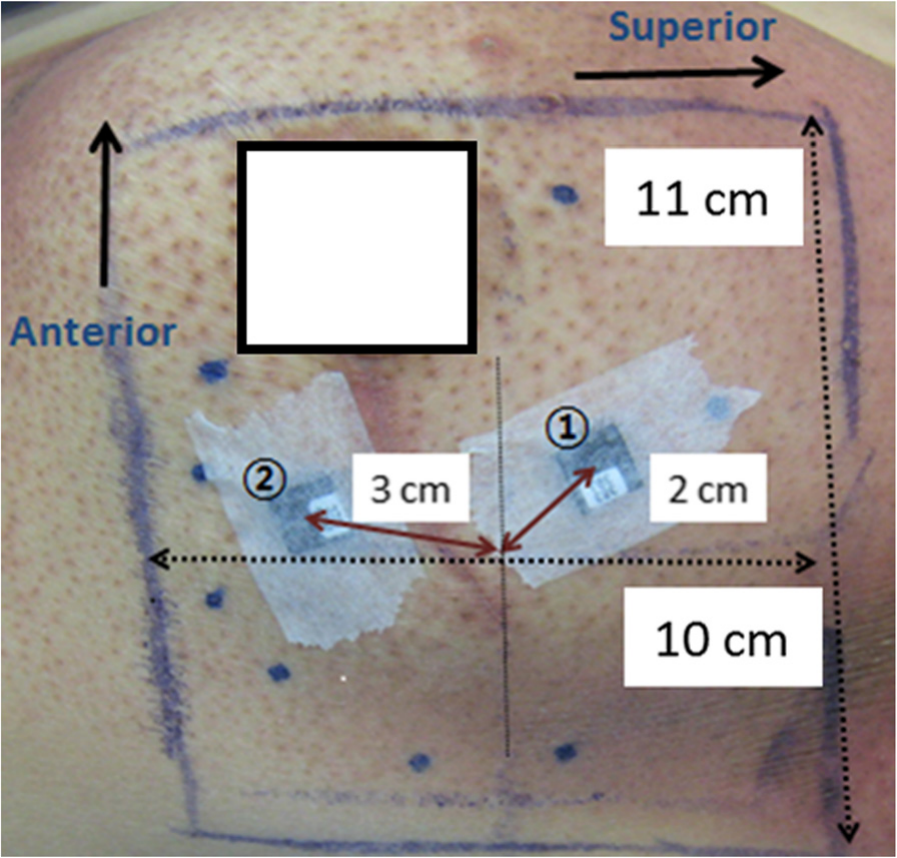
The setup picture of in-vivo dosimetry for patient 2. NanoDot™ optically stimulated luminescent (OSLD) dosimeters (Landauer Inc., Glenwood, IL) were used to measure the irradiated dose. The 15 × 15 cm^2^ cone was used to irradiate the target which size was 10 cm (superior-to-inferior) × 11 cm (Anterior-to-inferior). OSLD3 and OSLD4 are numbered 1 and 2 in the figure. OSLD3 and OSLD4 were located at the 2 cm and 3 cm from the isocenter, respectively

Calibrated OSLDs were in agreement within 2 % with dose readings of an ion-chamber at *d*_max_ of the 7 MeV. To minimize statistical uncertainty, five dose readings were performed and averaged over each dosimeter. In-vivo dosimetry was performed for the first three patients who were treated with the newly-developed 7 MeV beam. The setup of the OSLD was shown in Fig. 2 (patent 2). The treatment field was defined by a radiation oncologist on the basis of clinical examination. Monitor units were calculated using the in-house calculation software. Daily 180 cGy fractions were prescribed at *d*_max_. Two OSLDs were placed on the skin of each patient.

## Results

### Head component modelling for source parameters

**Fig. 3:**
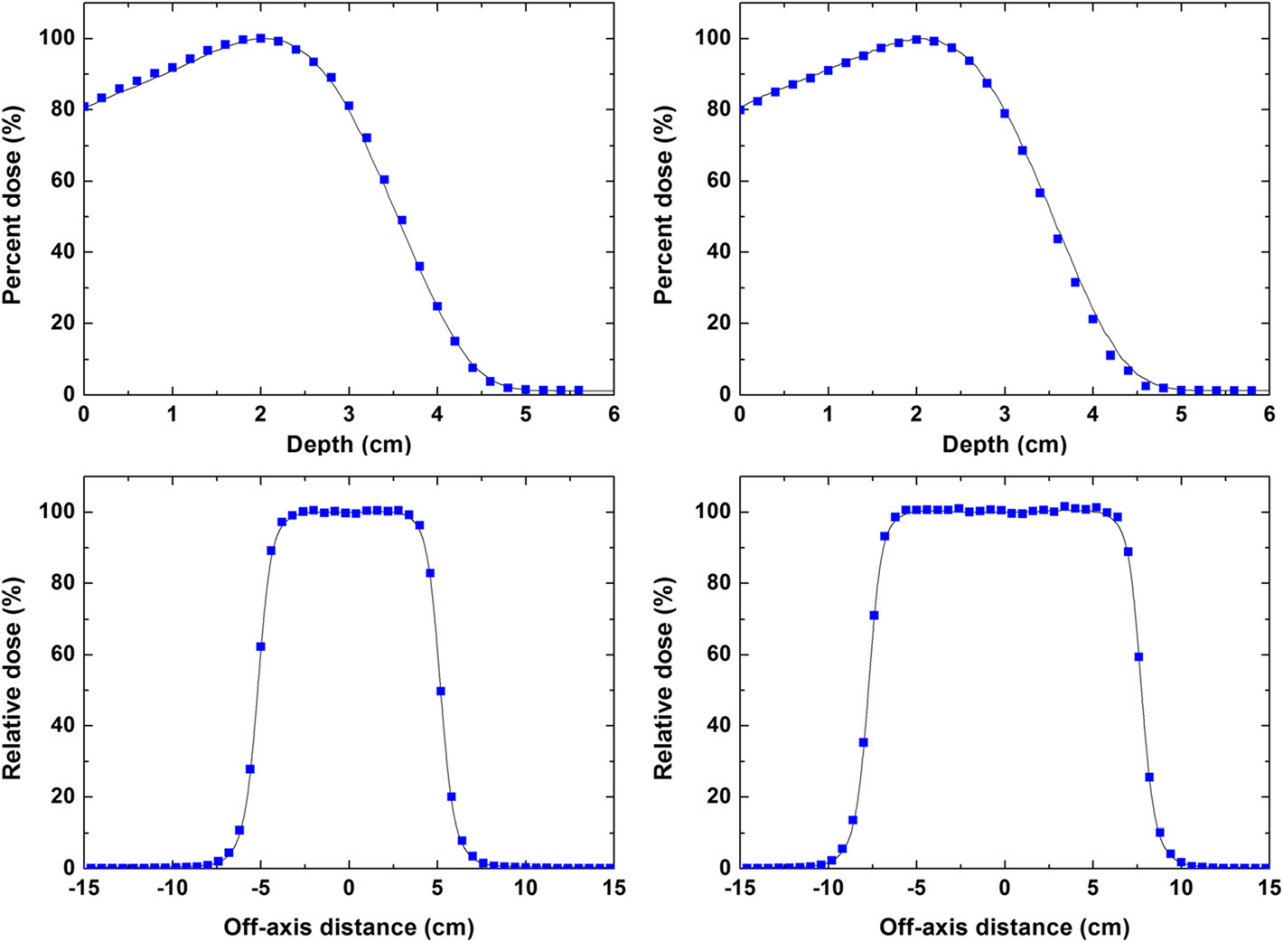
Comparison of the measured and calculated beam characteristics curves for 9 MeV beam. A comparison of the measured and calculated relative central-axis depth dose curves in water for 9 MeV beam of (**a**) 10 × 10 cm^2^ and (**b**) 15 × 15 cm^2^ cones. Measured and calculated cross-beam profiles at the depth of dose maximum in water for 9 MeV electron beam of (**c**) 10 × 10 cm^2^ and (**d**) 15 × 15 cm^2^ cones. Solid lines indicate measurements data with an ionization chamber (CC13). Squares indicate the calculated data from Monte Carlo simulations. The energy and lateral spread of incident electron beam for MC simulations were 9.85 MeV and 0.13 cm FWHM of Gaussian distribution, respectively

As shown in Fig. 3, the calculated values of *d*_*ma*x_, *R*_*50*_, and *R*_*p*_ agreed well with measured data, within 1 mm, when incident electron energy of 9.85 MeV was chosen. The calculated cross-beam dose profiles at *d*_*max*_ and *R*_*50*_ agreed well with measured data, within 2 %, except in penumbra regions when 0.13 cm FWHM of the incident electron beam was chosen. Thus, the energy and lateral spread of the incident electron beam for the standard 9 MeV electron beam were determined to be 9.85 MeV and 0.13 cm FWHM of Gaussian distribution, respectively. All subsequent simulations with the various energy degraders were carried out using these two source parameters.

### Monte Carlo simulations of the energy degraders

**Fig. 4:**
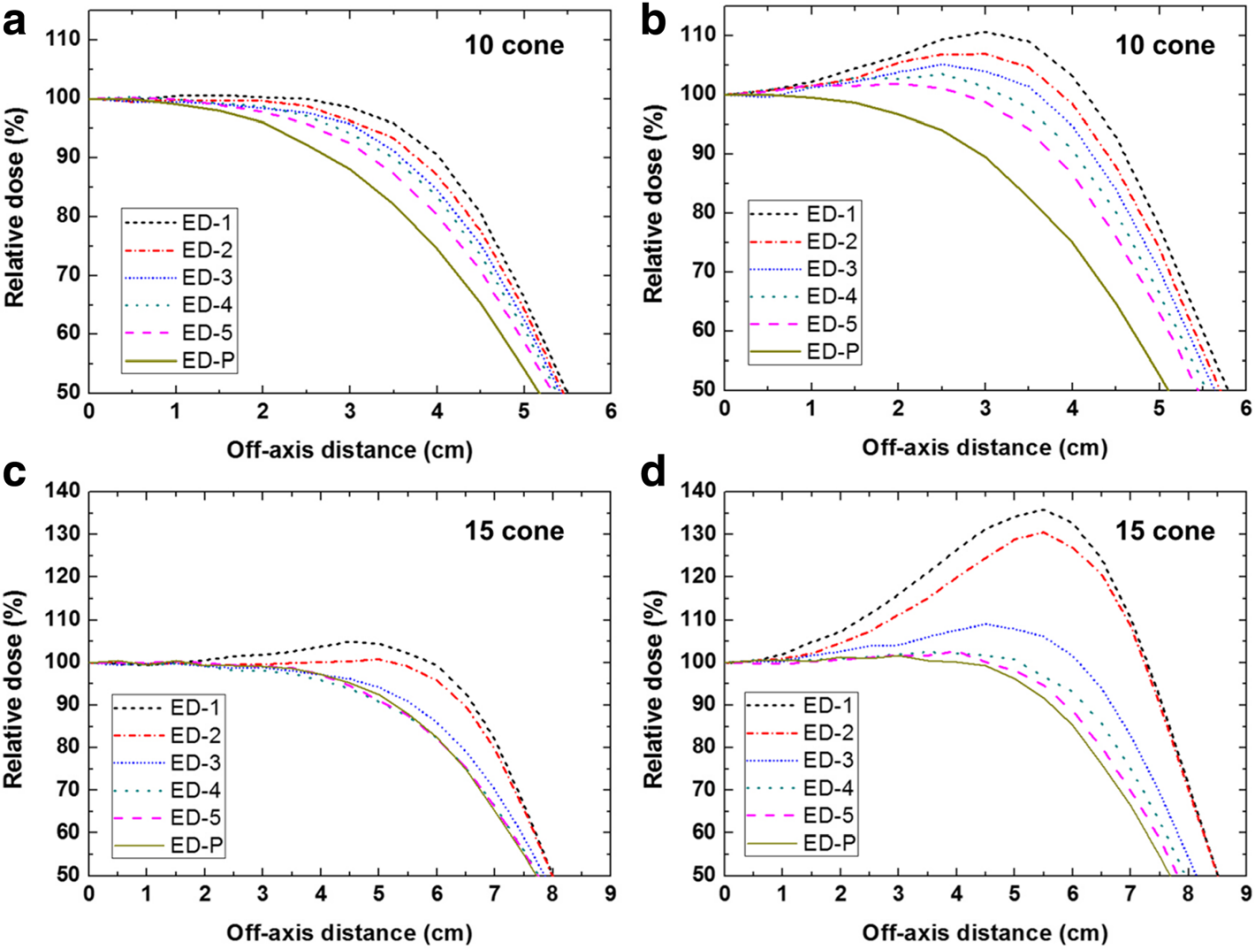
MC beam profiles of the 7 MeV electron beams for six designs. MC beam profiles of 10 × 10 cm^2^ cone were obtained (**a**) *d*_max_ and (**b**) *R*_*50*_ for six designs of the energy degrader (ED). MC beam profiles of 15 × 15 cm^2^ cone were obtained at (**c**) *d*_max_ and (**d**) *R*_*50*_ for six designs of the ED. An optimal design for 7 MeV beam was selected by considering uniformity of profile at *d*_max_ and *R*_*50*_. ED-4 was the final design for 10 × 10 cm^2^ and 15 × 15 cm^2^ cones

Calculated relative depth-dose curves along the central-axis were almost identical among the all ED designs described in Table 1. Therefore, dose profiles at *d*_*max*_ and *R*_*50*_ with the EDs were mainly used to evaluate the performance of the EDs. Figure 4 shows calculated cross-beam dose profiles along the x-axis normalized to the value at the central-axis. As shown in Fig. 4, the cross-beam dose profile of ED-P falls off rapidly as distance from the central axis increases. The ED-P failed to achieve acceptable profile uniformity.

For the 10 × 10 cm^2^ cone (Fig. 4a and b), electrons penetrating through the thick part of the ED degrade their energy more than those penetrating the thinner regions. Thus, truncated EDs show better uniformity with increasing *T*_*t*_ or decreasing *T*_*b*_, as compared to ED-P. Considering uniformity and the horn in the dose profiles at *d*_*max*_ and *R*_*50*_, ED-4 was selected as the optimal design for the 7 MeV beam of 10 × 10 cm^2^ cone. Fig. 4c and d for the 15 × 15 cm^2^ cone show the effects of the radius of the top layer (*r*) on the profile at *d*_*max*_ and *R*_*50*_, respectively. The horn dose at *R*_*50*_ increased as *r* decreased as shown in Fig. 3d. ED-1, ED-2 and ED-3 show unacceptable dose horns at 4 – 7 cm (>10 %). As ED-4 showed less penumbra region at *R*_*50*_ than did ED-5, ED-4 was selected as an optimized design for our 7 MeV beam of 15 × 15 cm^2^ cone.

### Relative central-axis depth-dose curve and cross-beam dose profiles

**Fig. 5:**
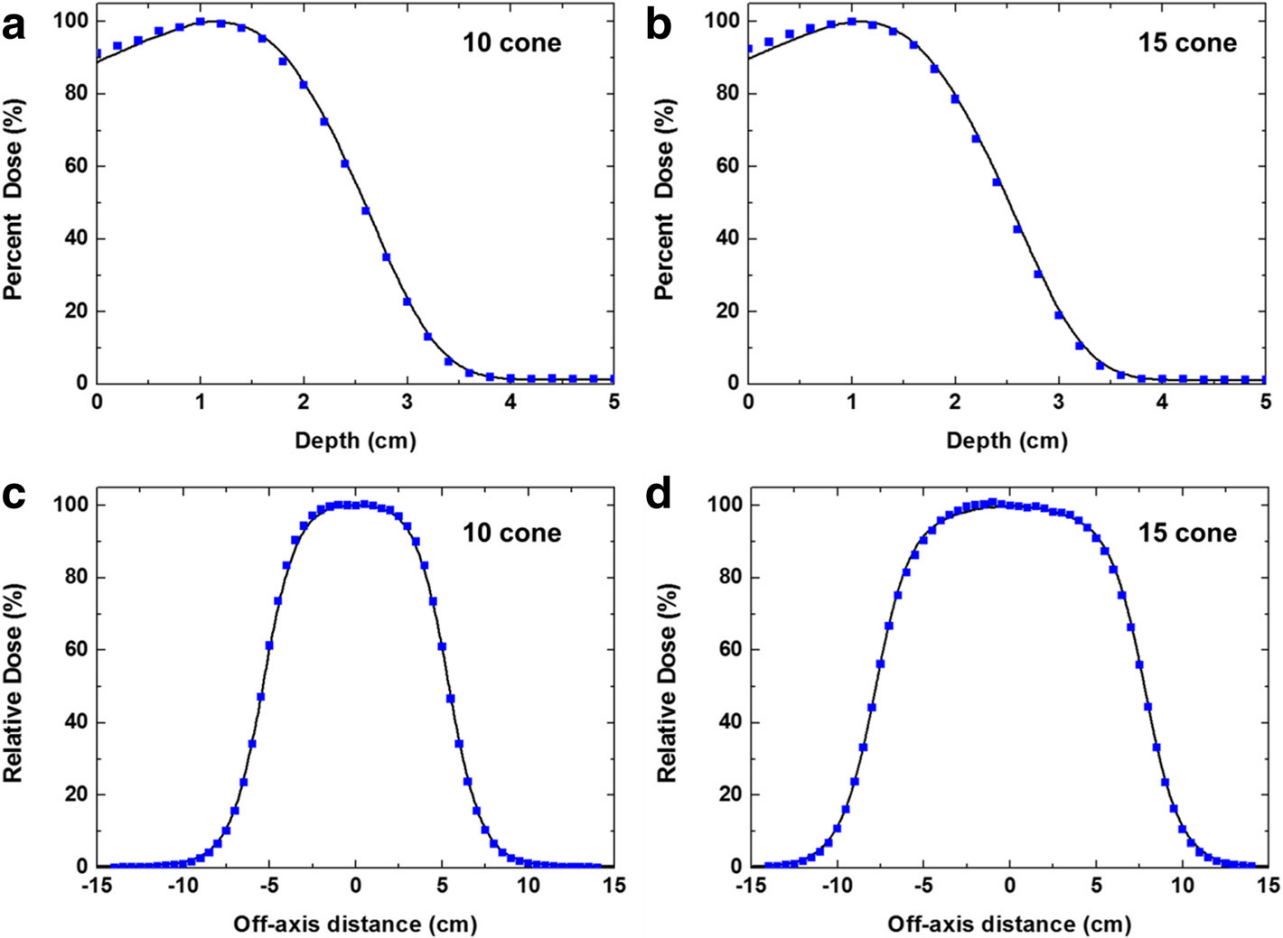
Comparison of the measured and calculated beam characteristics for new 7 MeV beam. A comparison of the measured and calculated relative central-axis depth dose curves in water for 7 MeV beam of (**a**) 10 × 10 cm^2^ and (**b**) 15 × 15 cm^2^ cones. Measured and calculated cross-beam profiles at the depth of dose maximum in water for 7 MeV electron beam of (**c**) 10 × 10 cm^2^ and (**d**) 15 × 15 cm^2^ cones. Solid lines indicate measurements data with an ionization chamber (CC13). Squares indicate the calculated data from Monte Carlo simulations

Based on the guidance of the previous section, the two optimized EDs for 10 × 10 cm^2^ and 15 × 15 cm^2^ cones were fabricated to produce 7 MeV electron beams. The relative central-axis depth-dose curves measured at 100 cm SSD are plotted in Fig. 5a and b along with the corresponding calculated values. The agreement between the calculations and measurements was found to be within 2–3 % at *d*_max_ for the two cone sizes employed in this study. The disagreement was larger in the region closer to the phantom surface for both cones. Cross-beam dose profiles of the 7 MeV beam at *d*_max_ are shown in Fig. 5c and d for 10 × 10 and 15 × 15 cm^2^ cones, respectively. All the measured profiles were in agreement with the calculated cross-beam dose profiles within 2–3 % except for the penumbra and beyond the penumbra.

**Fig. 6:**
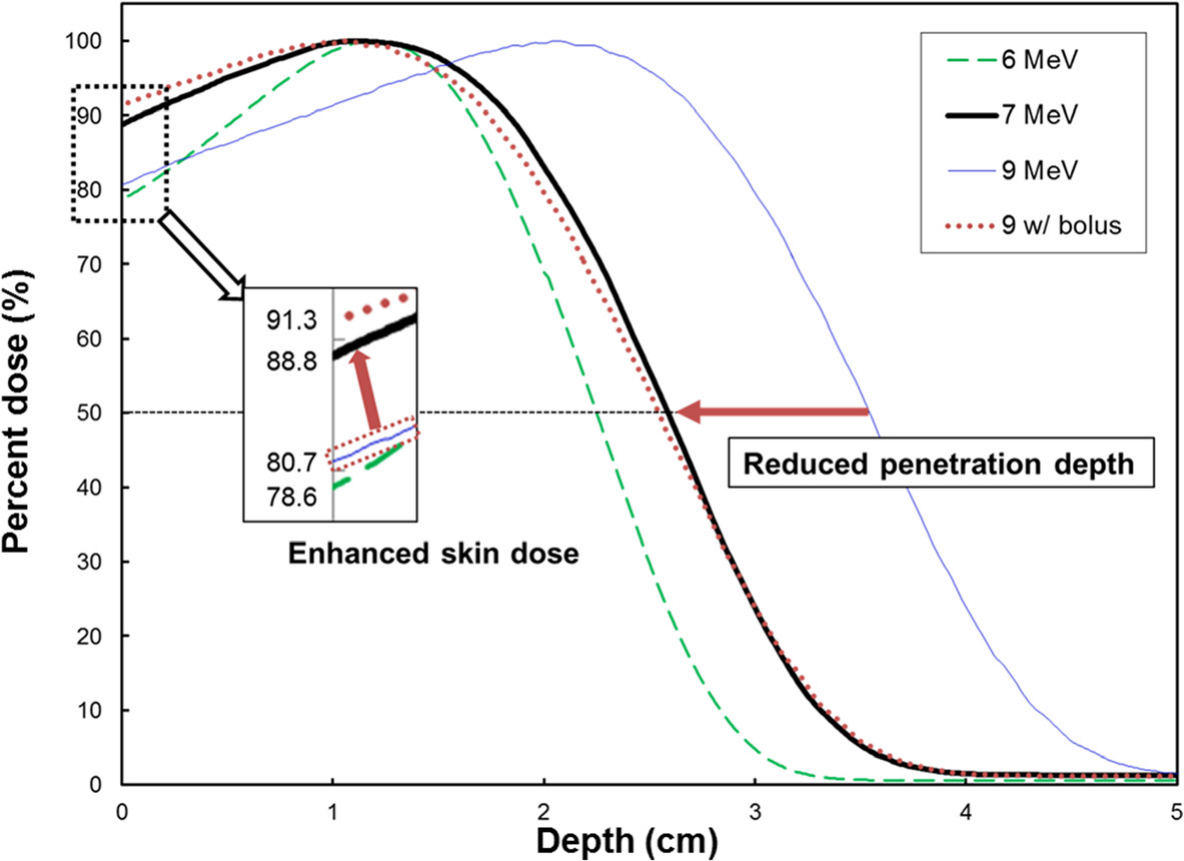
Comparison of measured depth-dose curves of electron beams. Comparison of measured depth-dose curves for new 7 MeV (bold solid), 9 MeV (solid), 6 MeV (dashed) and 9 MeV with 1 cm bolus (dotted) for 10 × 10 cm^2^ cone. Reduced penetration depth and enhanced skin doses for 7 MeV beam were shown in this figure

As shown in Fig. 6, the central-axis depth dose characteristics were compared with those of the standard 6 MeV beam and the 9 MeV beams with and without a 1 cm bolus. The characteristics of the 9 MeV beam with the bolus were similar to that of our 7 MeV beam, but had a surface dose a few % higher than the 7 MeV beam. The 7 MeV beam showed a reduced penetration to a depth between the 6 and 9 MeV beams, as well as an increased skin dose up to approximately 90 % of dose maximum. As shown in Tables 2 and 3, the practical range, *R*_p_, reduced from 4.39 to 3.35 cm for 10 × 10 cm^2^ cone and from 4.38 to 3.31 cm for 15 × 15 cm^2^ cone by using the 7 MeV beam instead of the 9 MeV beam. The values of *d*_max_ were 1.16 cm and 1.12 cm for the 10 × 10 and 15 × 15 cm^2^ cones, respectively.

**Table 2:** Central-axis depth dose characteristics for 10 × 10 cm^2^ cone. Central-axis depth dose characteristics for 7 MeV, 9 MeV, and 9 MeV with 1 cm bolus of 10 × 10 cm^2^ cone. *X*_con_ indicates X-ray contamination that is extracted from *R*_p_ plus 2 cm

Nominal energy (MeV)	Surface dose (%)	*d*_max_ (cm)	*R*_90_ (cm)	*R*_80_ (cm)	*R*_50_ (cm)	*R*_p_ (cm)	E_0_ (MeV)	*X*_con_ (%)
7	88.8	1.16	1.83	2.07	2.59	3.35	6.03	1.3
9	80.7	2.05	2.74	2.99	3.54	4.39	8.25	1.2
9 with bolus	91.3	1.05	1.74	1.99	2.54	3.33	5.92	-

**Table 3:** Central-axis depth dose characteristics for 15 × 15 cm^2^ cone. Central-axis depth dose characteristics for 7 MeV, 9 MeV, and 9 MeV with 1 cm bolus of 15 × 15 cm^2^ cone. *X*_con_ indicates X-ray contamination that is extracted from *R*_p_ plus 2 cm

Nominal energy (MeV)	Surface dose (%)	*d*_max_ (cm)	*R*_90_ (cm)	*R*_80_ (cm)	*R*_50_ (cm)	*R*_p_ (cm)	*E*_0_ (MeV)	*X*_con_ (%)
7	89.7	1.12	1.74	1.99	2.52	3.31	5.87	1.1
9	80.5	2.03	2.72	2.98	3.55	4.38	8.27	1.1
9 with bolus	91.1	1.03	1.72	1.98	2.55	3.32	5.94	-

### Output factors

The presence of the ED through the beamline caused a decrease in output due to the scattered electrons from the ED with large angles. Table 4 shows the calculated and measured output factors for the new 7 MeV beams. The reference of the output factor was measured using an open 10 × 10 cm^2^ cone of a 9 MeV beam, which has a value of 1.0. The measured output factors of the 7 MeV beam were 0.882 and 0.972 for 10 × 10 cm^2^ and 15 × 15 cm^2^, respectively. The measurements and calculations were within 1 % of each other for the 10 × 10 and the 15 × 15 cm^2^ cones. Table 5 shows the measured cutout factors of our 7 MeV beam for several different sizes of circular electron beam blocks of 10 × 10 cm^2^ and 15 × 15 cm^2^ cones. The cutout factor of the 9 MeV beam showed no significant variation, however the cutout factor of the 7 MeV beam widely varied with sizes of cutout inserts.

**Table 4:** Output factors for 7 MeV electron beam. Output factors for 7 MeV electron beam (reference: measured output for 9 MeV beam of 10 × 10 cm^2^ cone)

Cone (cm^2^)	Output factor calculated	Output factor measured	Difference (%)
10 × 10	0.888	0.882	0.6
15 × 15	0.980	0.972	0.8

**Table 5:** Measured cutout factors for 7 MeV electron beam. Measured cutout factors for 7 MeV electron beam (reference: measured output for 9 MeV beam of 10 × 10 cm^2^ cone; A/P: area to perimeter)

		A/P ratio									
Energy	Cone size (cm^2^)	0.75	1.00	1.25	1.50	1.75	2.00	2.25	2.50	3.00	3.75
7 MeV	10 × 10	0.335	0.490	0.623	0.738	0.805	0.858	0.870	0.882	-	-
	15 × 15	-	-	-	-	-	0.900	0.922	0.946	0.963	0.972
9 MeV	10 × 10	0.903	0.952	0.983	1.000	1.005	1.008	1.004	1.000	-	-
	15 × 15	-	-	-	-	-	1.003	1.005	1.007	1.004	0.996

### In-vivo dosimetry

Table 6 summarizes the results of in-vivo dosimetry for three patients in different irradiation conditions. In Table 6, the differences are expressed as the measured dose relative to the expected surface dose (160 cGy) on the central axis. We assume that the expected value at the surface dose was 89 % of the maximum dose. Differences between measured and expected doses were less than 8 cGy except in the case of one OSLD. The deviation of OSLD 4 could be explained by its large off-axis distance (approximately 4 cm) and thus a prolonged SSD due to the curved skin.

**Table 6:** In vivo dose results. In vivo doses of 7 MeV beam measured with optically stimulated luminescence dosimeters

	OSLD number	SSD (cm)	Cone size (cm^2^)	Measure Dose (cGy)
Patient 1	1	100	10	156
	2			153
Patient 2	3	100	15	152
	4			139
Patient 3	5	105	15	152
	6			152

## Discussion

We developed energy degraders for a new electron energy for the breast boost irradiation when using the electron mode of a commercial LINAC. Iterative MC simulations were performed to obtain the optimal structure and dimensions of the EDs. By placing the ED in the lowermost scraper of the applicator to minimize scattered doses out of the treatment field, it was also secured during LINAC rotation without an extra fixing device. The design of EDs can be considered as one of the alterative of modifying the LINAC’s own energy when we need certain electron beam between energies. Thus, the developed method can be easily applied to other intermediate energies by selecting the base design of this study which consists of a truncated cone attached on top of a plane plate.

A Lucite® plate was shown to be a suitable material for the reduction of X-ray contamination.^33^ Scattered electrons from the ED that interact with the applicator are able to cause X-ray contamination. Since the X-ray contamination of electron beams is produced mainly in the scattering foils of the LINAC [43, 44]. The slight increase (0.1 %) in the X-ray contamination of depth-dose curves of the 7 MeV beams was most likely caused by the ED. The Lucite® ED in place can be used to raise the surface dose of a 9 MeV electron beam to up to 90 % of dose maximum as well as to reduce the penetration depth of electrons. Thus, the technique suggested here could be an acceptable alternative to the use of a bolus for boost treatment of the tumour bed of the breast. In particular, the ED minimizes the variation in patient’s setup. Also the setup time is reduced when the ED is implemented, as the device is located on the lowermost scraper of the electron applicator rather than on the patient’s skin. As shown in Fig. 6, this technique is able to fill the energy gap between 6 MeV and 9 MeV to some degree.

The tantalum wire mesh bolus and metal bolus increased the surface dose and kept only a small change of the depth dose curve [4, 7]. These materials should be placed on the patient surface. It may cause the setup variations during the treatment course. However, the final design of the ED is easily fixed to the lowermost scarper of electron applicator at the same position for every fraction as shown in Fig. 1a.

The results of the in-vivo dosimetry showed lower than the expected value on the central axis (see Table 6). The measured points on the skin were several cm away from the central axis as shown in Fig. 2. Since our EDs were designed to achieve optimal uniformity at *d*_max_, the cross-beam dose profile at shallow depth was somewhat parabolic from the center to the penumbra, rather than uniform. Furthermore, the measured skin doses could vary with off-axis distances due to varying SSDs caused by the slope of the breast. These reasons may contribute to the lower in-vivo results than the expected value. Nonetheless, all in-vivo doses to the skin ranged from 78 to 87 % of the prescription dose.

Unfortunately, current treatment planning systems (TPS) do not provide adequate flexibility to support beam data from a modified electron spoiler or ED on the electron applicator. Thus, the 3D dose distribution with the EDs was not calculated by the TPS. An in-house Monte Carlo-based TPS is under development to evaluate the 3D dose distribution of the 7 MeV beam.

## Conclusions

In this study we developed novel energy degraders for 7 MeV electron beams that are capable of reducing the penetration depth and enhancing the skin dose, compared to the standard 9 MeV beam. The efficacy of the optimally-designed EDs was validated through experimental evaluation. Thus the optimally designed ED in the 9 MeV beamline provides breast conserving patients with a new energy option of 7 MeV for boost of the shallow tumor bed. It would be an alternative to bolus and thus eliminate inconvenience and concern about the daily variation of bolus setup.

## Abbreviations

ED: Energy degrader
PDD: Percent depth dose
PTV: Planning target volume
OAR: Organ at risk
LINAC: Linear accelerator
eMLC: Electron multi-leaf collimator
FLEC: The few leaf electron collimator
MC: Monte Carlo
SSD: Source to surface distance
FWHM: Full width at half maximum
PMMA: Polymethly methacrylate
TPS: Treatment planning system
*d*_max_: Depth of dose maximum
*R*_90_: 90 % of dose maximum
*R*_80_: 80 % of dose maximum
*R*_50_: 50 % of dose maximum
*E*_0_: The most probable energy of the incident electron beam
*X*_con_: X-ray contamination that is extracted from *R*_p_ plus 2 cm
*T*_t_: Top layer thickness
*T*_b_: Bottom layer thickness
*r*: The radius of the top layer
EP-P: A simple plate design of ED
A/P: Area to perimeter
